# Application of case-based learning in psychology teaching: a meta-analysis

**DOI:** 10.1186/s12909-023-04525-5

**Published:** 2023-08-25

**Authors:** Fanghui Wu, Tao Wang, Danxu Yin, Xiaoxiao Xu, Cancan Jin, Nan Mu, Qingrong Tan

**Affiliations:** Department of Basic Psychology, School of Psychology, Army Medical University, Chongqing, China

**Keywords:** Case-based learning, Traditional lecture, Psychology, Education, Meta-analysis

## Abstract

**Background:**

Case-based learning (CBL) has been found to be effective for many subjects, but there is currently a lack of evidence regarding its utility in psychology education. The present study investigated whether CBL pedagogy can improve students’ academic performance in psychology courses compared to the traditional teaching methods.

**Methods:**

A systematic review and meta-analysis were conducted to investigate the effectiveness of CBL in psychology teaching. Databases including *PubMed, Embase, Web of Science, China National Knowledge Infrastructure (CNKI)*, the *VIP database*, and *Wanfang data* were searched to find eligible randomized controlled trials. Pooled effect estimates were calculated using Hedges’ g under the random effects model, and a subgroup analysis was carried to investigate the heterogeneity among studies.

**Results:**

Fifteen studies with 2172 participants, 1086 in the CBL group and 1086 in the traditional lecture-based teaching group, were included in the meta-analysis. Students in the CBL group scored significantly higher on exams than those in the lecture-based group [Hedges’ g = 0.68, 95%CI (0.49, 0.88), *p* < 0.00]. Relatively high heterogeneity was noted among the included studies. Publication bias was examined by the funnel plot and Egger’s test, but did not significantly influence the stability of the results. A subsequent evaluation using the trim-and-fill method confirmed that no single study was skewing the overall results. A qualitative review of the included studies suggested that most students in the CBL group were satisfied with the CBL teaching mode.

**Conclusions:**

This meta-analysis indicated that the CBL pedagogy could be effective in psychology education, and might help increase students’ academic scores, while encouraging a more engaging and cooperative learning environment. At present, the application of CBL in psychology education is in its initial stage. Problems related to the curriculum itself, research methodology, and challenges faced by both teachers and learners have confined its practice. Fully tapping into the strengths of CBL in psychology teaching will require additional work and advancing research.

## Background

Case-based learning (CBL), also known as case-based teaching or case-based instruction, is a student-centered pedagogy that requires students’ active participation in analyzing and discussing cases provided by the teacher. Although no standardized definition has been acknowledged, the teaching method is thought to have been systematically introduced by the Harvard Law School in the early twentieth century, and is a signature teaching format in the Harvard Business School. Their website claims that under the CBL method curriculum, students need to “put themselves in the shoes of actual decision-makers” to solve the problem by using what they have learned in advance [1]. The successful application of CBL in the fields of law and business has promoted its application in other types of professional education, such as medicine, dentistry, and science education, as an active and important pedagogy.

Compared with traditional lecture-based strategies, CBL has some special advantages and characteristics. The CBL approach has previously been described in terms of its aim(s), content, and processes. In brief, the primary aim of CBL is to prepare students for professional practice [2]. That is, the use of cases empowers students to apply theoretical knowledge to contextual situations, and thus facilitates knowledge transfer, as well as problem-solving and critical thinking skills [2, 3]. Authentic and effective teaching cases are the key content of CBL. The achievement of CBL objectives largely depends on the case construction and facilitation. Kim et al. [4] proposed a conceptual framework wherein cases must be relevant, realistic, engaging, challenging, and instructional to be effective. Moreover, cases are often situation-based, and can be delivered in multiple ways such as the text (the most common way), the computer or web assisted format, and the real-life simulation. Some have stressed on the combined use of case-based method and the situation-based learning in diverse forms such as role play [5], while we believe that case analyses shall be the core in the teaching process. Inquiry-based learning is regarded as the best instruction process for this type of case-based learning [2], but this may be done in different ways. In general, group discussion is the most common application. It has been pointed out [6, 7] that case discussion is the most important part as the process, because engaging and debriefing in case discussions can help students obtain new knowledge, connect new knowledge to experience, and build their knowledge structure [2]. Therefore, CBL excels in linking theory to practice since students are exposed to and then asked to solve the real or simulated cases deliberately composed by the teaching team. Another method of inquiry-based education, problem-based learning (PBL), is often compared with CBL. What distinguish CBL are that CBL format requires students’ prior knowledge to solve specific problems in the profession, the process of which is under the guidance of the trained teachers. By comparison, when using PBL, learning occurs during solving the problem with little previous knowledge or the teacher’s control of the whole class, and thus there would be more “unfocused tangents” [8]. In this perspective, CBL is more structured, effective, and accepted by students and the teaching faculty especially at the undergraduate education. Moreover, case-based instruction benefits both students and teachers in that it stimulates the students’ learning motivation and enthusiasm, and it urges teachers to constantly refresh their professional knowledge and boost innovation [9]. In this regard, applying CBL in teaching brings challenges and higher expectations to both teachers and students.

Psychology is an applied discipline, which includes educational psychology, clinical psychology, organizational psychology, and others. As an important component of medicine and health systems, psychology is essential for both psychology majors and future clinicians. Graduates are expected to use psychological knowledge to deal with the relationship between doctors and patients, provide health care suggestions and prescribe psychotherapies if necessary. At the same time, the subject features strong theoretical bases that are intricate to understand. However, traditional lecture-based teaching is somewhat an indoctrination of theories, while CBL gives attention to both theory and application. In this case, a growing number of teaching teams have realized the advantages of using case-based instruction to teach psychology.

Since the 1990s, CBL has been increasingly implemented in psychology courses, including educational psychology, clinical psychology, introductory psychology and so on. Research on the application of CBL in teaching psychology has mainly focused on the content and processes related to using it, such as course planning, case construction and facilitation, and in endeavors to more efficiently implement CBL. From the teaching perspective, it is critical to ensure the optimal development and best use psychological cases. Based on the overall aims and requirements of the course syllabus, the instructor or teaching team may emphasize the construction of the content and structure of the case, utilizing mature cases or creating specific model cases to support and bridge the gap between theory and practice [10–13]. Meanwhile, the strategy, concrete methods and steps required to utilize cases in psychology courses is another vital topic, especially for prospective teachers in the training program and as teaching practice included in various professional courses [14, 15].

With regard to the students’ learning, improvements in academic performance and the development of self-teaching, clinical reasoning, and satisfaction in learning are the core concerns [11, 16]. Some positive and valuable feedback about the effectiveness of CBL in psychology teaching have been obtained in previous studies [11, 16]. However, several challenges associated with applying CBL in psychology teaching also exist, such as the lack of viable cases, insufficient time for successful implementation, students’ lack of readiness to utilize the new information, and so on. Moreover, the effects of CBL on academic performance (as indicated by exam scores) are still being debated because the method is often unstructured and subjective compared to the traditional lecture-based teaching [10], some students appreciated the lecture-based method in helping them prepare for a written exam [17]. Although several studies have proved its effectiveness, some research showed that there were no significant differences in the final exam scores between students in a CBL group and a traditional lecture-based group [18], so additional work is needed on this topic. Therefore, this study applied a meta-analysis to determine the effectiveness of CBL in teaching psychology courses, and to identify the factors influencing the efficacy of the method, so as to have a more thorough understanding of the strategy and to better support subsequent studies of the use of CBL in psychology teaching.

## Methods

### Study design

The meta-analysis adhered to the guidelines of the Preferred Reporting Items for Systematic Reviews and Meta Analyses (PRISMA statements) [19].

### Literature search

The research data were collected from open-access journals from both China and abroad. To be specific, the literature search was carried out in the *PubMed, Embase, Web of Science, China National Knowledge Infrastructure (CNKI)*, and *VIP databases*, and using *Wanfang data* from the earliest publication date (in December of 1976) to August 29th, 2022.

The key terms used in search were as follows: “case-based learning”, “case-based”, or “CBL” and “psychology”. Filters were used to seek out target randomized controlled trials.

### Inclusion and exclusion criteria

The study adopted the following inclusion criteria: (1) randomized controlled trials with an experimental group involving the CBL pattern and a controlled group receiving traditional lectures in psychology-related courses; (2) participants were undergraduates or vocational school students; (3) students’ academic performance was quantified by the exam score that was reported with a mean and standard deviation. Studies were excluded when (1) different research methods (other than RCTs) were used; (2) the study was missing data; (3) the publication was a review or meta-analysis. For missing data, we meant that studies did not report the number of participants, the exam scores with a mean and standard deviation nor could these indices be calculated, or any other data that were essential for data synthesis.

For the quality evaluation of the included studies, all of them were first graded by 2 authors according to the standards of the Jadad scale [20] concerning randomization, double blinding, and withdrawals and dropouts, which is normally applied for clinical experimental research studies. Specifically, for randomization, if words such as *randomly, random*, or *randomization* were used, the study was given 1 point, and 1 additional point was given for specifying the method and it being used in an appropriate way. Double blinding also increased the total score by 2 points, 1 for mentioning the method, and an additional 1 point for the adequacy and specific description of the method. For withdrawals and dropouts, 1 point was given if there were no withdrawals/drop-outs, or if it was clearly stated that there were participants who withdrew or dropped out of the study. The highest possible full score was 5, so a total score of 1–2 indicated a low-quality article while a score of 3–5 refers to a high-quality article.

However, some scholars have put forward different opinions [21, 22] about the use of randomized experiments in education research, and suggested that blinding both the teacher and the student could be impossible [23]. For this reason, the scores of the Jadad scale may not adequately indicate the quality of the studies included in the meta-analysis. To our knowledge, no evaluation tool has been developed specifically to assess the quality of these types of studies in the educational field.

### Data extraction and literature screening

All the searched articles were managed with the EndnoteX9 software. Two researchers worked independently for record screening and data extraction under the guidance of the inclusion and exclusion criteria. If there were controversies during the screening process, the studies were discussed until the two researchers reached a consensus. For the qualified papers, information concerning the author, publication year, sample size, majors of participants, teaching methods used, courses taught, and outcome data were collected. The outcome data comprised quantitative statistics of the students’ academic scores, as well as their satisfaction and evaluation of CBL if this information was available.

### Statistical analysis

Statistical analyses were conducted using the STATA 16.0 software. Continuous variables were demonstrated as the standardized mean difference (SMD), and the results were based on the 95% confidence interval (CI). First, the effects of CBL on the participants’ academic performance was examined by pooling the extracted data together using the effect size of Hedges’ g. The heterogeneity of the data was investigated using the *I*^*2*^ value. If *I*^*2*^< 50% and *p* > 0.1, a fixed effects model would be used, otherwise a random effects model would be chosen for the meta-analysis. In addition to heterogeneity testing, subgroup analyses were performed and a Galbraith plot was generated. Lastly, publication bias was evaluated by conducting the Egger’s test and using a funnel plot. If publication bias was detected, a trim and fill method would be applied to see whether the bias related to a specific publication would influence the results.

## Results

### Study characteristics

**Fig. 1:**
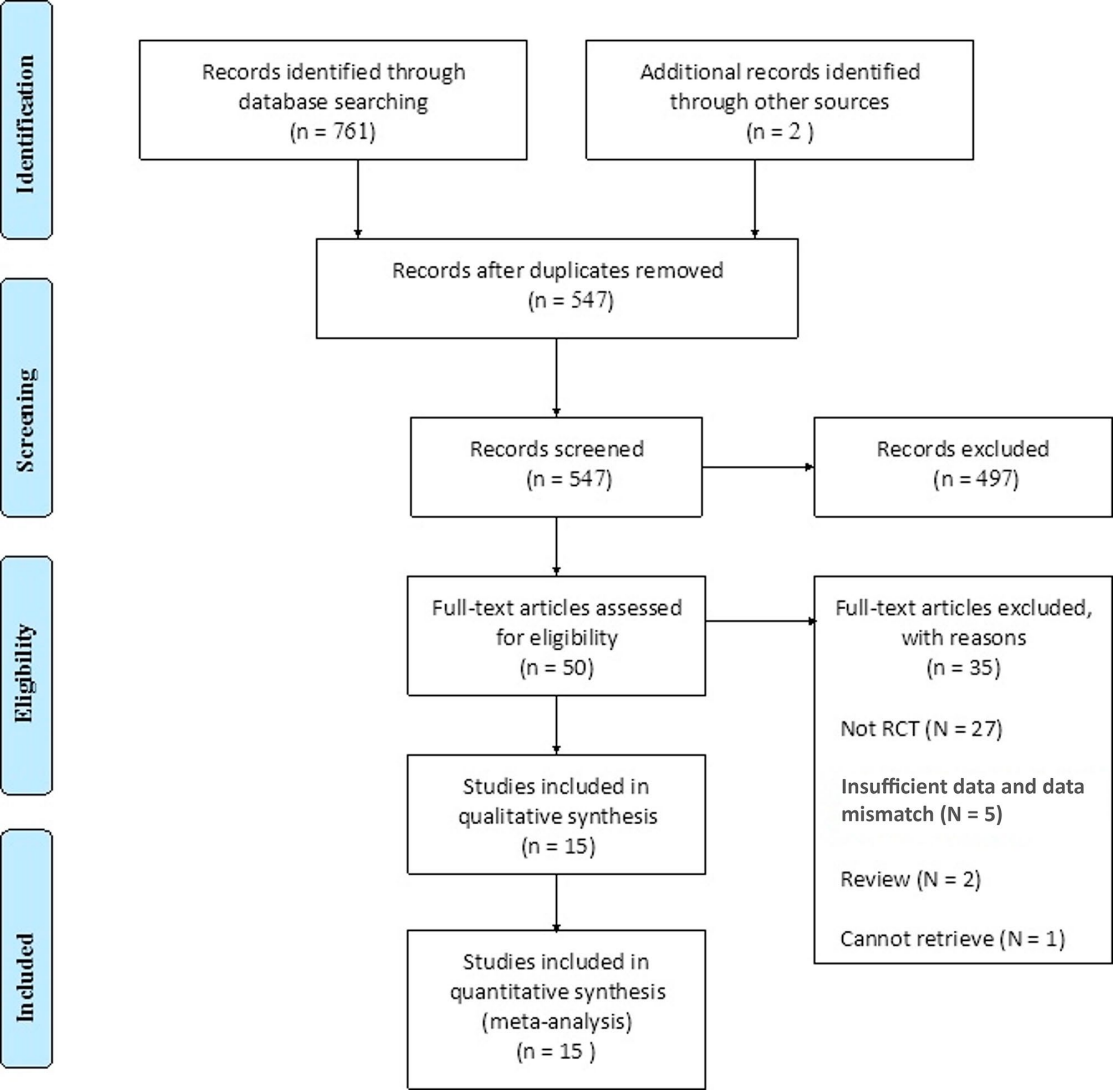
The PRISMA flow chart for the selection of studies included in the meta-analysis

The systematic search of the literature identified 763 relevant articles from online databases (Fig. 1). Specifically, 143 records were obtained from *CNKI*, 191 records from *Wanfang Data*, 105 records from *VIP databases*, 152 records from *PubMed*, 134 records from *Embase*, 36 records from the *Web of Science*, and 2 additional records were obtained from other sources. The EndNoteX9 tool helped remove 169 duplicate records automatically, and 47 additional duplicates were excluded manually. After screening the titles and abstracts to exclude irrelevant studies, 50 records were kept for further review. Of the 50 articles, 35 studies were removed after the full-text was read for various reasons: 27 studies did not follow the design of randomized controlled trials, five studies reported insufficient data and data mismatch, two studies were reviews, and the full text version of one study could not be retrieved.

Finally, 15 studies [11, 16, 18, 24–35] in total were included in the meta-analysis and further discussions, and the characteristics of each study are shown in Table 1. The 15 studies comprised 2172 participants, 1086 in CBL groups and 1086 in control groups. Among them, five studies compared the effects of CBL with traditional lectures in medical psychology courses, five studies involved trials in teaching nursing psychology, and the other five studies were carried out in teaching management psychology, the instruction for a clinical internship, experimental psychology, introductory psychology, and the psychology of adjustment. Although these courses are fundamental in psychology education, each type of curriculum follows a different pattern. We predict that there would be a certain level of heterogeneity due to the variety of courses, and subgroup analyses were done based on the different course types.

**Table 1:** Characteristics of the included 15 RCT studies

Author, year	Major	Course	Teaching methods	Academic performance				Student’s evaluation or attitudes towards CBL
				Experimental group		Control group		
				N	Scores (Mean ± SD)	N	Scores (Mean ± SD)	
Mayo, 2002	Not mentioned	Introductory psychology	CBL/LBL	70	83.23 ± 10.29	66	76.76 ± 12.43	Students held largely positive attitudes towards CBL: (1) more than 65% of students found the method helpful; (2) more than 64% of students found the cases realistic.
Mayo, 2004	Not mentioned	Psychology of adjustment	CBL/LBL	64	84.69 ± 9.73	58	75.07 ± 11.17	Students held largely positive attitudes towards CBL: (1) more than 75% of students found the method helpful; (2) most students found the method triggered their interests and involvements to a more challenged learning.
Li et al.,2010	Nursing	Nursing psychology	CBL / LBL	159	82.70 ± 7.30	163	79.80 ± 8.20	N/A
Song, 2010	Nursing	Nursing psychology	CBL + simulated teaching/ LBL	40	89.85 ± 6.82	40	81.85 ± 5.94	Students held positive attitudes towards CBL and believed that CBL had improved their: (1) comprehensive competence (92.5%), (2) self-confidence (75.0%), (3) clinical coping skills (95.0%) and ability to apply knowledge (97.5%).
Xie & Li, 2013	Nursing	Nursing psychology	CBL / LBL	80	88.48 ± 6.24	80	81.34 ± 10.71	CBL method was conducive to strengthen nurses’ mental fitness.
He, 2014	Public health management	Medical psychology	CBL / LBL	45	82.67 ± 18.88	44	74.09 ± 18.09	Students held significantly different attitudes between CBL and LBL: (1) CBL is more effective than LBL to complete the teaching objectives; (2) CBL brought better interaction between teachers and students, stimulated learning enthusiasm and initiative, and improved reasoning and practices.
Hou & Jing, 2014	Nursing	Nursing psychology	CBL / LBL	118	75.58 ± 10.08	90	78.36 ± 11.36	CBL method helped enhance students’ self-study ability.
Kong & Zhou, 2014	Applied psychology	Management psychology	CBL / LBL	43	86.00 ± 6.00	47	79.00 ± 12.00	Most students believed that CBL triggered their interests and initiatives in learning, and it enhanced communication and cooperation.
Zhang et al., 2014	Not mentioned	Medical psychology	CBL / LBL	63	76.33 ± 6.44	61	71.49 ± 6.54	N/A
Luo, 2017	Clinical medicine	Medical psychology	CBL / LBL	143	84.27 ± 5.12	149	79.92 ± 6.37	CBL could deepen students’ understanding of knowledge, promote knowledge transfer, arouse interests, and enhance problem solving ability.
Pei et al., 2017	Nursing	Nursing psychology	CBL + simulated teaching / LBL	102	85.8 ± 12.7	98	77.3 ± 8.4	N/A
Su et al., 2019	Clinical medicine	Medical psychology	CBL / LBL	30	86.70 ± 5.32	31	80.49 ± 5.10	N/A
Song et al., 2019	Applied psychology	Psychology internship	CBL / LBL	18	91.30 ± 4.70	18	82.30 ± 7.70	Most students believed that cases were inspiring that could trigger their interests and enhance problem solving ability.
Nie et al., 2021	Clinical medicine	Medical psychology	CBL + TBL / LBL	80	78.39 ± 7.08	100	74.38 ± 8.43	More than 90% of the students were satisfied with CBL and TBL which might help improve communication and teamwork, while 11.3% of the students did not adapt to the teaching mode.
Xiao, 2021	Applied psychology	Experimental psychology	CBL + PBL + Flipped classroom / LBL	31	82.2 ± 6.62	41	74.7 ± 12.63	Mostly were satisfied with the teaching mode.

The risk of bias for the included randomized controlled trials was assessed according to the Jadad score [20], and the results are presented in Table 2. In terms of randomization, nine studies complying with a randomization process without explaining the specific methods used received a score of one, and one study [32] using lot drawing was scored two. No study applied double-blinding methodology in the design. No withdrawals or dropouts were seen in any of the trials, so all were given a score of one. Thus, one study scored three points, nine scored two points, and the other five scored one point in the quality assessment.

**Table 2:** Quality assessment of the 15 included RCT studies using the Jadad scale

Author, year	Randomization	Double blinding	Withdrawals & dropouts	Total score
Mayo, 2002	Not specified, 1	Not described, 0	No W&D, 1	2
Mayo, 2004	Not specified, 1	Not described, 0	No W&D, 1	2
Li et al.,2010	Not specified, 1	Not described, 0	No W&D, 1	2
Song, 2010	Not described, 0	Not described, 0	No W&D, 1	1
Xie & Li, 2013	Not specified, 1	Not described, 0	No W&D, 1	2
He, 2014	Not specified, 1	Not described, 0	No W&D, 1	2
Hou & Jing, 2014	Not specified, 1	Not described, 0	No W&D, 1	2
Kong & Zhou, 2014	Not described, 0	Not described, 0	No W&D, 1	1
Zhang et al., 2014	Not described, 0	Not described, 0	No W&D, 1	1
Luo, 2017	Not described, 0	Not described, 0	No W&D, 1	1
Pei et al., 2017	Not specified, 1	Not described, 0	No W&D, 1	2
Su et al., 2019	Lot drawing, 2	Not described, 0	No W&D, 1	3
Song et al., 2019	Not specified, 1	Not described, 0	No W&D, 1	2
Nie et al., 2021	Not specified, 1	Not described, 0	No W&D, 1	2
Xiao, 2021	Not described, 0	Not described, 0	No W&D, 1	1

Regarding outcome variables, besides academic performance (indicated by means and standard deviations), seven studies also investigated CBL’s effects through questionnaires such as teaching evaluations, self-assessments of learning abilities, psychological scales, and so on. Generally, students expressed positive attitudes towards the case-based approach and provided positive feedback. More details can be seen in Table 1.

### Quantitative analysis of pooled effects

**Fig. 2:**
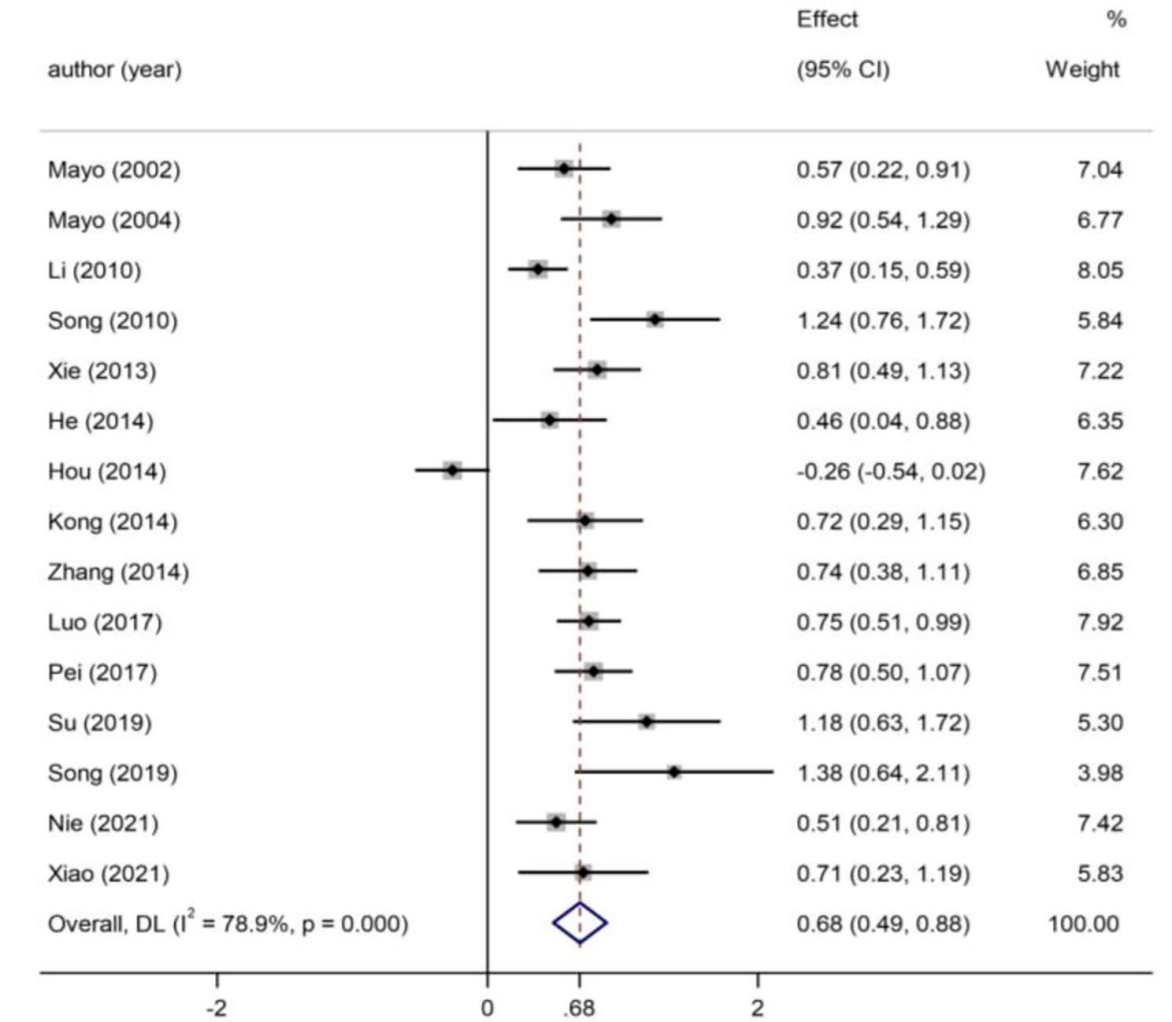
Forest plot showing the impact of CBL on students’ academic performance compared with conventional LBL under the random effects model

A statistically significant pooled effect size (Hedges’ g) was observed when comparing students’ academic scores based on whether they had CBL or traditional lecture-based teaching. As shown in the forest plot (Fig. 2), the total effect of CBL yielded better results than lecture-based learning [Hedges’ g = 0.68, 95%CI (0.49, 0.88), *p* < 0.00]. The heterogeneity was relatively high (*I*^*2*^= 78.90%, *p* < 0.00), suggesting that a random effects model should be applied.

### Investigation of heterogeneity

**Fig. 3:**
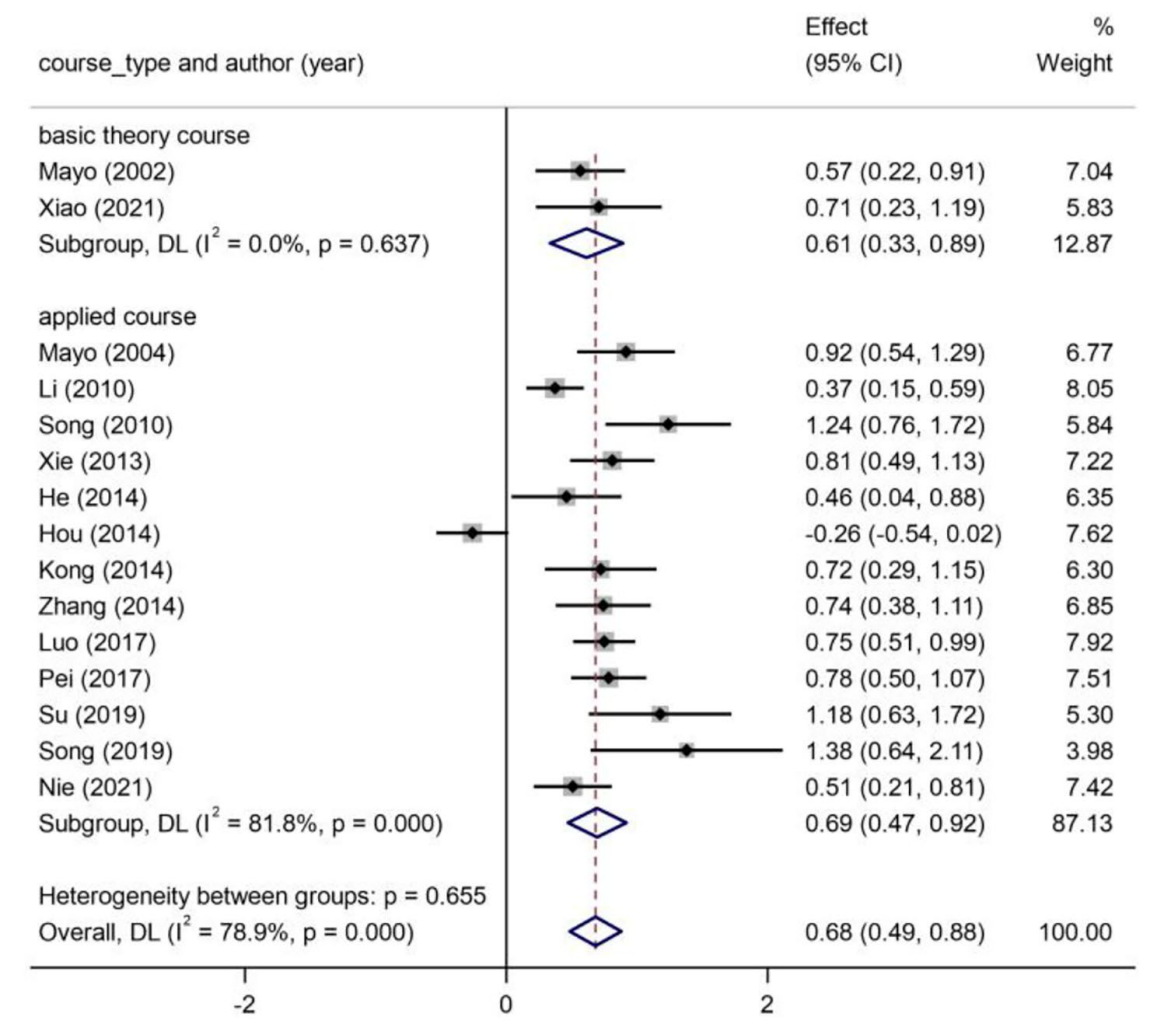
Forest plot for the subgroup analysis based on different course types

**Fig. 4:**
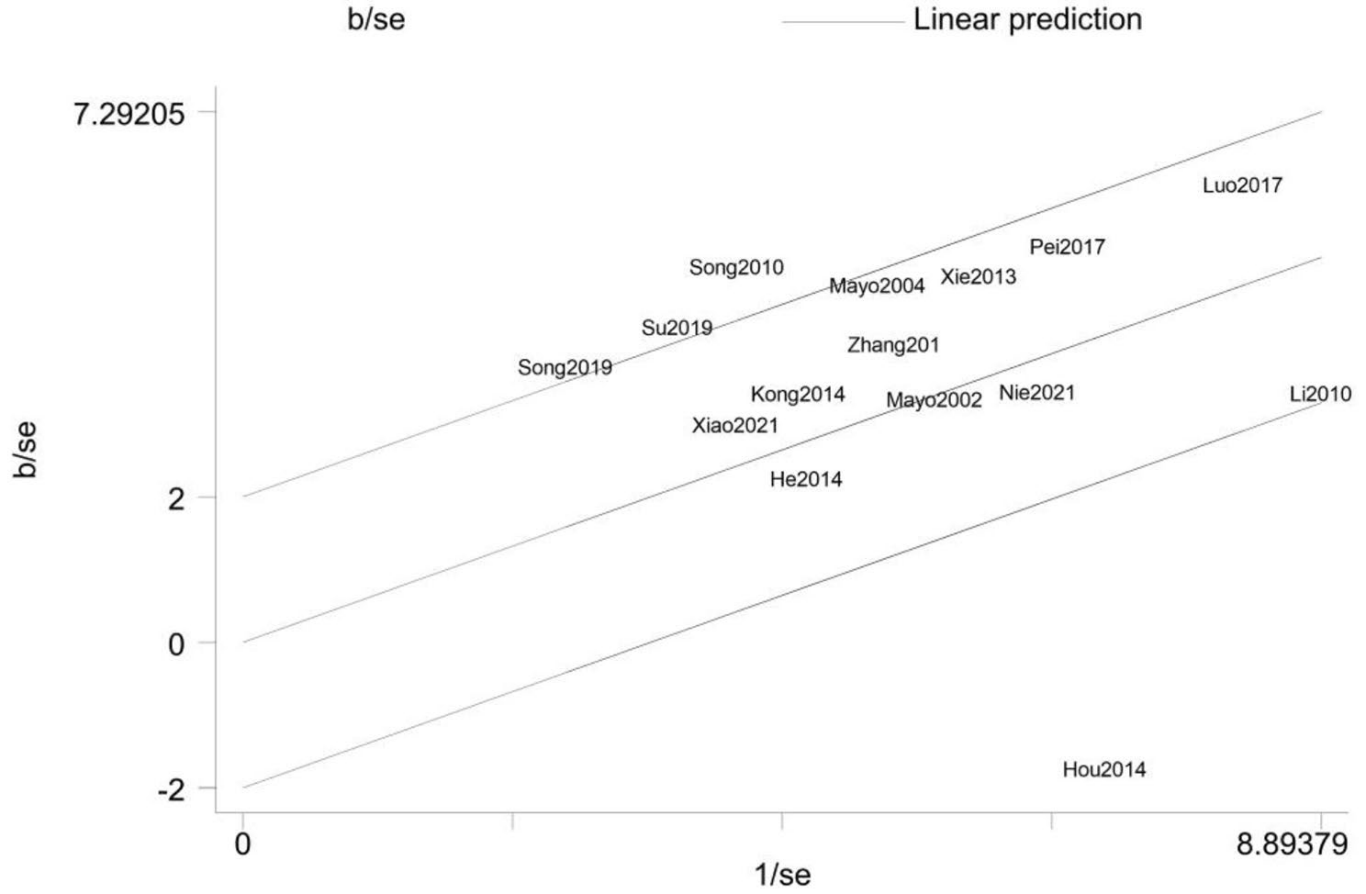
Galbraith plot of the 15 studies

Due to a high heterogeneity across the meta-analysis, a subgroup analysis was conducted to explore the source of the heterogeneity. We considered that the characteristics of the different courses may have resulted in high heterogeneity. Thus, we divided the 15 studies into two categories based on the different course types: basic theory courses (n = 2) [11, 35] and applied courses (n = 13) [16, 18, 24–34]. The results presented in Fig. 3 suggest that in basic theory courses, the students in the CBL group had significantly higher exam scores than did those in the control group [Hedges’ g = 0.61, 95%CI (0.33, 0.89), *p* < 0.00]. Likewise, in the applied psychological courses, the exam scores in the CBL group were obviously higher than those in the traditional lecture group [Hedges’ g = 0.69, 95%CI (0.47, 0.92), *p* < 0.00]. Hence, the results of the subgroup analysis suggested that CBL led to significant improvements in scores related to teaching both types of psychology courses. After this sub-grouping, the heterogeneity in the basic theory group became non-significant (*I*^*2*^ = 0.00%, *p* = 0.64), while it remained high in the applied course group (*I*^*2*^ = 81.80%, *p* = 0.00), suggesting that the different course types may not be the source of heterogeneity. This finding led us to further investigate the studies using a Galbraith plot (Fig. 4).

**Fig. 5:**
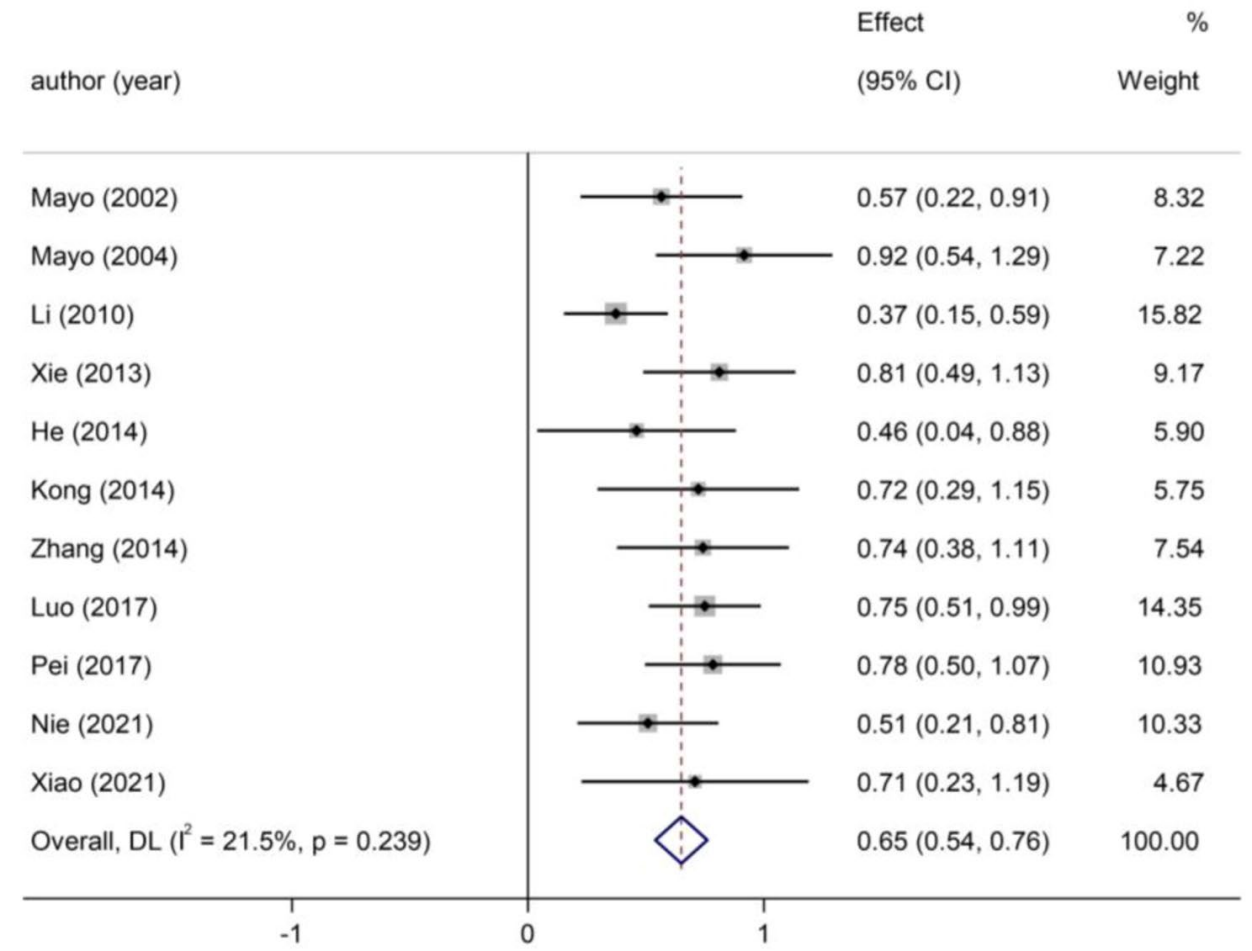
Forest plot after removing four studies [18, 26, 32, 33]

As shown in Fig. 4, four studies [18, 26, 32, 33] outside the parallel lines were considered to be the potential sources of heterogeneity. After removing them, no obvious heterogeneity was found across studies (*I*^*2*^= 21.50%, *p* = 0.24). However, even after removing these four studies, a forest plot (Fig. 5) indicated that the total examination scores of students in the CBL group were still significantly higher than those in the control group [Hedges’ g = 0.65, 95% CI (0.54, 0.76), *p* < 0.00].

### Sensitivity analysis

**Fig. 6:**
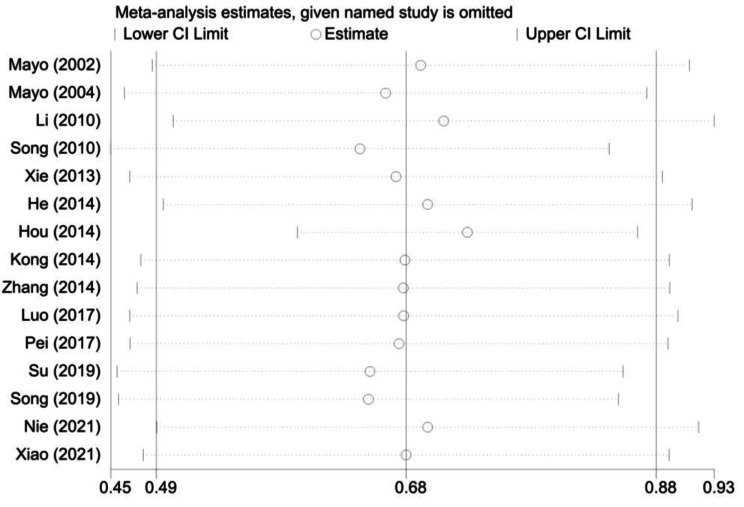
Sensitivity analysis of the pooled studies

A sensitivity analysis was performed by omitting studies one-by-one to see whether any single study could affect the statistical significance of the results in the meta-analysis. Figure 6 shows that the point estimate of pooled effects after removing each study always stayed within the confidence interval, suggesting that the results were stable.

### Publication bias

**Fig. 7:**
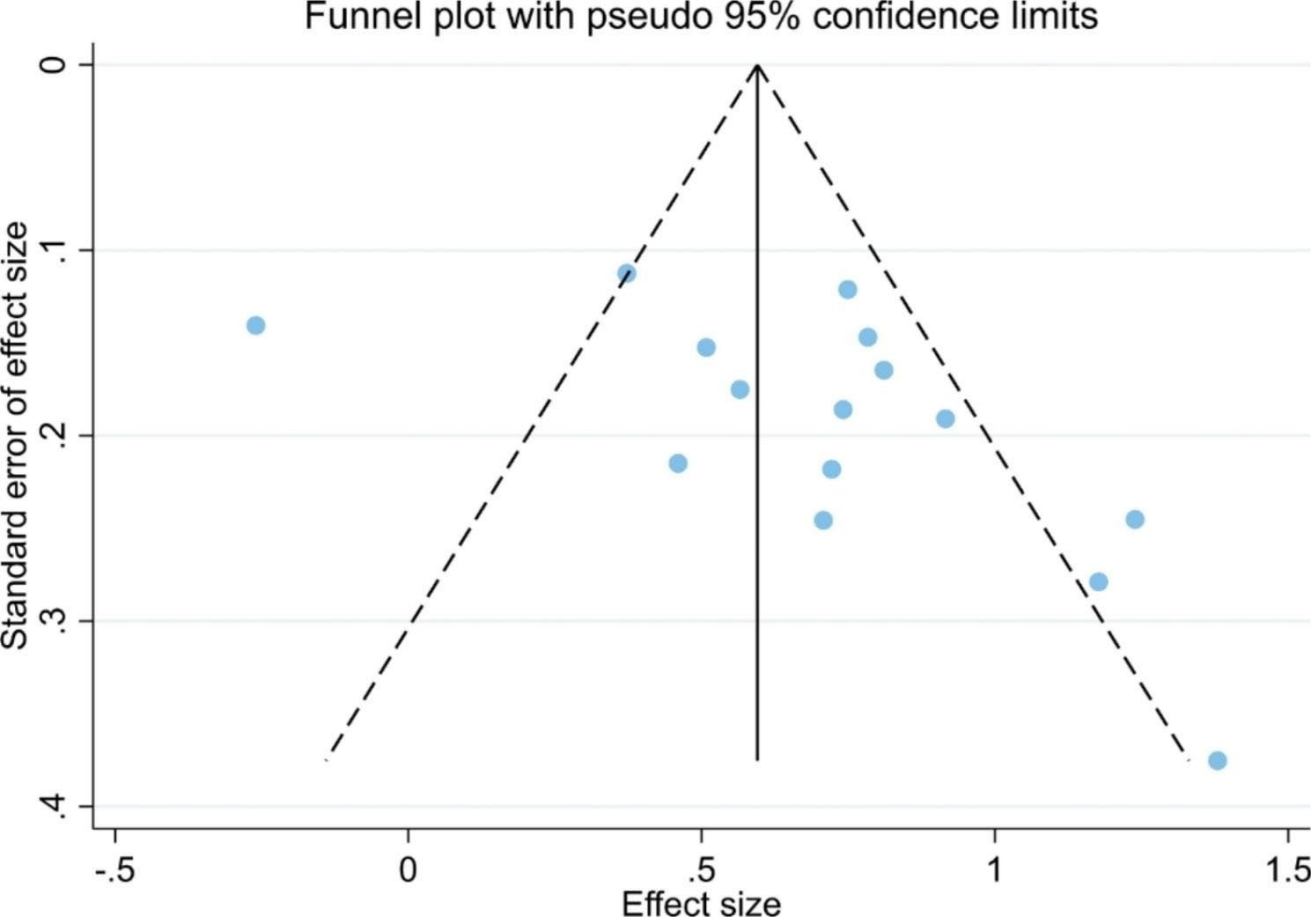
Funnel plot of the studies included in the meta-analysis

To further assess the data, a funnel plot was generated. The funnel plot (Fig. 7) appears to be somewhat asymmetrical. Egger’s test was also performed as a reference, and the findings suggested that there was slight publication bias in the meta-analysis (*t* = 2.21; *p* = 0.046).

**Fig. 8:**
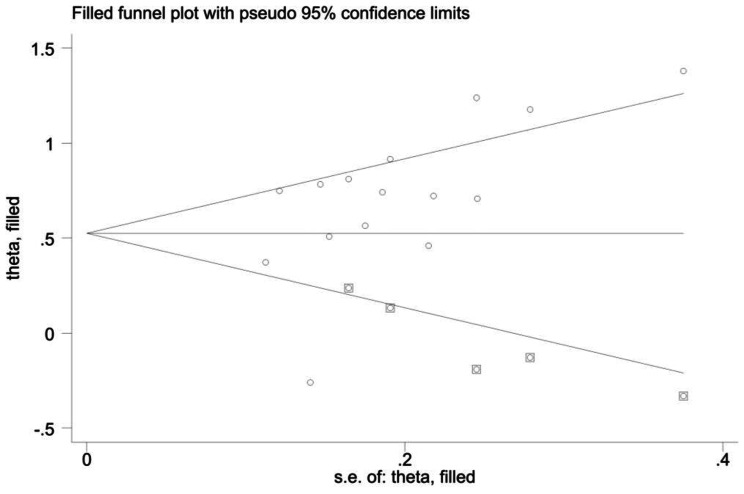
Results of trim-and-fill method

Due to the publication bias indicated by Egger’s test, a trim-and-fill method was applied to test whether the bias could affect the results. Figure 8 indicated that after hypothetically filling five missing studies, the funnel plot would become visually symmetrical, wherein no publication bias would exist. Notably, the new pooled effect under the random effects model was still significant [Hedges’ g = 1.69, 95%CI (1.41, 2.04), *p* < 0.00], indicating that the existence of publication bias did not significantly influence the robustness of the meta-analysis.

### Qualitative evaluation of the effectiveness of CBL

Eleven studies reported results from questionnaires and open-ended surveys that investigated the students’ evaluation of and attitudes towards CBL after experiencing the whole teaching process. Table 1 displays these descriptions in detail. All eleven studies reported that CBL was beneficial for students, and its beneficial effects included: (1) aroused learning interests [16, 24, 28, 30, 33]; (2) enhanced self-study abilities [16, 18]; (3) improved communication and cooperation [16, 28, 34]; and (4) improved problem-solving capabilities [30, 33].

## Discussion

Students studying psychology often find themselves in a dilemma since they are expected to apply what they have learned in their future career or in real life to settle problems and make decisions, while the abstract theories they have learned are divorced from reality. It is difficult to provide these skills via only traditional lectures. CBL has been proposed as a method to fix this gap. Based on a literature search, we found that most of the published studies were qualitative, and there have been only a few quantitative studies designed to examine the strengths of CBL in psychology education, and there have been even fewer studies with high-quality experimental designs. Among the limited RCT studies, the CBL teaching mode was more often implemented in applied courses, especially in medical psychology and nursing psychology. The two courses are interdisciplinary that applies psychological theories and techniques into clinical and health-care practice. Thus, CBL is more often practiced in teaching these applied psychological courses. Evidences of CBL’s effectiveness in other psychology courses are fewer. That is to say, the application of cases-based method in teaching psychology is still in its early stages. Thus, we aim to gather data from the existing literature to investigate whether CBL could be useful in psychology education by meta-analysis. To our knowledge, this is the first meta-analysis conducted to investigate the effectiveness of CBL in psychology education. Our results support the idea that CBL could be beneficial in teaching psychology and may be superior to traditional lecture-based learning. Students in the CBL group acquired higher scores in examinations and improved their problem-solving and critical thinking, which is in agreement with other studies in clinical medicine education [36] and dental education [37].

Several factors may affect the efficacy of CBL in psychology teaching. For instance, the way the teaching team organize the cases, the quality of the teaching staff, the duration of case analysis, and many others may affect the successful implementation of CBL. Importantly, the quality of the case is critical for eliciting the students’ interest [10], thus promoting substantive case discussions [13]. The cases from the included studies were from clinical practice or fictional, but few provided this information, so the importance of whether the case was ‘real’ and many other details about the case could not be analyzed. While we believe fictional cases can be as effective as true clinical cases for CBL, it is necessary to ensure that the fictional cases are believable and indistinguishable from real cases.

The strategy that the instructor uses in facilitating and debriefing class discussions is also important [10]. The comparative research reported by Engle and Faux [13] suggests that a higher percentage of contribution to case discussions was observed in the classes where more authority was offered to the student by the instructor, while more structured instruction could facilitate the stronger use of psychological theory. It is difficult to determine which strategy is superior, since each instructor has his or her own style and customs, and student learning styles also vary. However, regardless of the teaching style, it is important that instructors receive training in CBL teaching before its implementation to ensure their professionalism and to increase efficacy of the case instruction. Students’ responses also affect the instructor’s implementation of CBL. One study on students’ stress levels found that the students’ stress escalated when they changed to a CBL environment from traditional teaching [38]. A study by Baeten et al. [39] suggested that gradually introducing students to CBL is important for their autonomous motivation and academic achievement. Thus, it is suggested that instructors should keep an eye on students’ feelings and obtain timely feedback during the implementation of case instruction.

The meta-analysis showed relatively high heterogeneity among the studies. The subgroup analysis showed that different course types did not appear to account for this heterogeneity. However, we believe that this result could offer some implications. Psychology curriculum is broad and extensive that courses vary from each other. Just as the saying goes, one man’s meat maybe another man’s poison, we wondered whether CBL could still be statistically more effective than traditional LBL in different types of psychology courses especially in those theory-oriented courses. The results supported that CBL could be more effective than LBL on improving students’ academic scores whenever teaching the courses of the basic theories or the application content. Nevertheless, the basic theory courses group only comprised two courses, indicating that CBL is more often practiced in those application-oriented courses. It suggests that we could make the effort to use case-based method in theory-oriented psychology courses in the future.

To further investigate the source of heterogeneity and whether it will influence the stability and reliability of the result, a Galbraith plot was drawn. It helped identify four studies [18, 26, 32, 33] that may have contributed most to the heterogeneity. The heterogeneity may be related to differences in the research design, the implementation of the intervention as discussed above, as well as the characteristics of participants. For instance, Song et al. [33] applied CBL to teach the mental outpatient internship that students learnt from hands-on cases by observing the process of psychotherapy at the scene, and after the observation, students would hold a discussion and try to simulate the scene under the guidance of the instructor. This first-hand experience of cases differs from the in-class discussion that not only students’ previous knowledge would be recalled, but also their emotions and feelings would be elicited. It is in line with the Constructivist views of learning that it is essentially social in nature [10]. This diverse type of case-based teaching mode might cause heterogeneity statistically, but we think it is worth trying in other applied psychology courses teaching in the future. It is difficult to analyze all of these elements using the limited information offered by each article, but we tried to elaborate on the methodology of the included RCTs. Specifically, the participants in the study of Su et al. [32] all knew the information about the trial which may cause biases since the students in the CBL group might feel that they were paid special attention. Likewise, Song [26] used non-probability sampling which might cause heterogeneity. There are different views regarding the use of controlled experiments when performing education research. Some have stressed the importance of diversity in educational research methods, believing that double-blinding is hard to follow even in the simpler experiments performed outside the educational setting [21]. Moreover, randomization may also be difficult to achieve because the student participants had been streamed beforehand based on different programs. It is recommended that in the education field, evaluators should rely more on ethnographic methods with more descriptive information [22], such as that obtained by observing or videotaping. Nevertheless, an RCT may still be possible in the education field. However, it is necessary to design more effective trials or experiments to balance the internal and external validity with regard to the unique characteristics of education research.

The qualitative evidence drawn from questionnaires and surveys of the 11 studies suggests that a large percentage of students were more strongly motivated to learn when CBL was applied. Overall, the students’ subjective evaluations suggested that CBL helped their learning reach a deeper level, which means that they had a better and more extensive understanding of the theoretical knowledge being taught in the course. Additionally, because CBL is a team effort, the students’ communication and cooperation skills would noticeably be strengthened during the class, based on both interactions between teacher and student and interactions between and among students.

Nevertheless, some limitations exist in this study. First, few studies were included in the meta-analysis, resulting in a relatively small sample size. We adopted the inclusion criteria used for randomized-controlled trials, leading to the exclusion of qualitative studies and any study with another design. In addition, education research may be inevitably subject to biases caused by the implementation of blinding and randomization [22]. It is suggested that the type of educational intervention, control environment and the instructors’ training experiences, which are not assessed as part of the Jadad score, should be investigated when evaluating the reliability of education research [22]. Educational research is currently very diverse, which is why we wished to collect and synthesize more evidence to provide more reliable results using a meta-analysis. Second, the effectiveness of CBL was mainly quantified based on the academic score, which is simple to use, but shows only short-term results, which may be relatively subjective. The efficacy of CBL should also be evaluated using other educational indicators, such as motivation and interest, and it should be tracked in the long-term. Third, the study only compared the effects of CBL with LBL. In the future, CBL should be compared with other new teaching methods to further investigate its strengths and weaknesses.

## Conclusion

In general, the present meta-analysis indicates that CBL is more effective than LBL in improving students’ academic scores in psychology, and was a method welcomed and appreciated by the students. CBL features the combination of theories and practice, makes the classroom more engaging and encourages more cooperation and communication among students and teachers. In this way, the students’ learning interest would be stimulated, and their problem-solving and critical thinking skills could be better practiced. However, the application of CBL in psychology education is still in its initial stage, and there are no systematic standards or acknowledged rules to follow. Quantitative research on this area is still limited, with a lack of appropriate methods to control for probable biases to reach better decisions about the effectiveness of CBL in teaching psychology. Hence, the application of CBL in psychology teaching must be the subject of further research and exploration.

## Data Availability

The datasets used and analyzed during the current study are available from the corresponding author upon reasonable request.

## Declarations

**Ethics approval and consent to participate**.

**Consent for publication**.

### Author’s information

^1^Department of Basic Psychology, School of Psychology, Army Medical University, Chongqing, China.

## List of abbreviations

CBL: Case-based learning
LBL: Lecture-based learning
TBL: Team-based learning
PBL: Problem-based learning
